# Injectable Biomaterials for Dental Tissue Regeneration

**DOI:** 10.3390/ijms21103442

**Published:** 2020-05-13

**Authors:** Håvard Jostein Haugen, Poulami Basu, Mousumi Sukul, João F Mano, Janne Elin Reseland

**Affiliations:** 1 Department of Biomaterials, Institute of Clinical Dentistry, Faculty of Odontology, University of Oslo, 0317 Oslo, Norway; poulami1986@gmail.com (P.B.); mousumi.sukul@odont.uio.no (M.S.); j.e.reseland@odont.uio.no (J.E.R.); 2 CICECO – Aveiro Institute of Materials, Department of Chemistry, University of Aveiro, 3810-193 Aveiro, Portugal; jmano@ua.pt

**Keywords:** hydrogel, injectable, scaffold, pulpal regeneration

## Abstract

Injectable biomaterials scaffolds play a pivotal role for dental tissue regeneration, as such materials are highly applicable in the dental field, particularly when compared to pre-formed scaffolds. The defects in the maxilla-oral area are normally small, confined and sometimes hard to access. This narrative review describes different types of biomaterials for dental tissue regeneration, and also discusses the potential use of nanofibers for dental tissues. Various studies suggest that tissue engineering approaches involving the use of injectable biomaterials have the potential of restoring not only dental tissue function but also their biological purposes.

## 1. Injectable Biomaterials for Dental Tissue Engineering

This is a narrative review, which discusses the state of the science of injectable biomaterials for dental tissue engineering from a theoretical and contextual point of view. Biomaterials, which can be either synthetic, natural and pure or composite materials play an important role in dental tissue engineering to interface with surgical procedures and cell biology [1,2], and also include injectable biomaterials which can serve as stem cell carriers and supramolecular hydrogels, as well as in situ gelling hydrogels [3,4,5]. The modern design of micro-carriers can accomplish biodegradability, biocompatibilities, as well as offer required features for improving biological responses [2,6,7,8].

Injectable scaffolds play an important role in tissue regeneration since they provide numerous advantages over pre-formed scaffolds [9] and are used for regeneration of craniofacial and dental tissues, which consist of alveolar bone, temporo-mandibular joint, periodontal ligament, dentin, and pulp [5,10,11,12]. Various studies [13,14,15,16] have concluded that tissue engineering approaches involving the use of biomaterials replace missing or damaged craniofacial and dental structures, as well as restore their biological purposes.

## 2. Classification of Hydrogels

**Figure 1:**
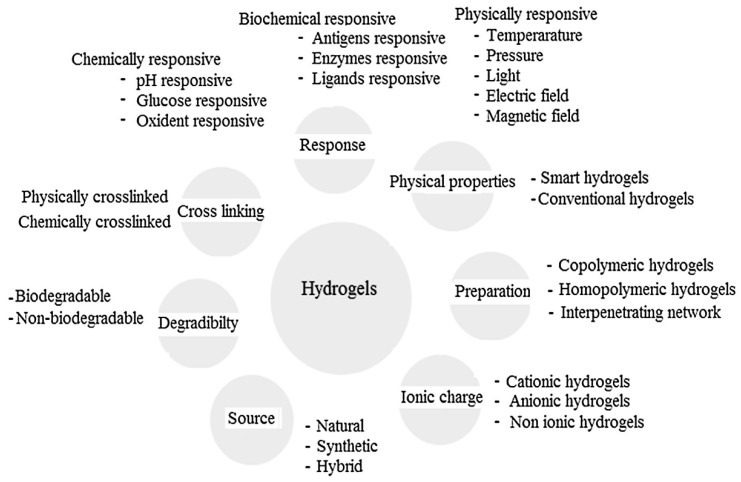
Hydrogels based on the different properties such as source, physical properties, crosslinking, ionic charge, response and degradability Reprinted from [17] with permission from Elsevier.

Hydrogels are broadly classified based upon their original sources, preparation method and physico-chemical behaviour, such as crosslinked, interpenetrating network, chemical and stimuli responsive and protein-based hydrogels, as depicted in Figure 1. This review provides a brief insight of the classification of prominent hydrogel systems which have attracted biomaterials scientists over a past few years.

Conventionally, based upon crosslinking, hydrogels are classified into two distinct categories: physically crosslinked and chemically crosslinked hydrogels [17].

### 2.1. Physically Crosslinked Hydrogels

Hydrogels prepared by physically crosslinked methods are usually reversible gels and are relatively easy to produce without any crosslinking agents during synthesis process. The crosslinking agents may affect the integrity of components (cells/proteins) entrapped into the hydrogel system and need to be removed before hydrogel application. Moreover, hydrogels formed with non-covalent bonds may be moldable, permitting a better defect filling ability. This leads to an increased demand of physically crosslinked hydrogels in the field of biomaterials and tissue engineering [18].

### 2.2. Chemically Crosslinked Hydrogels

The chemically crosslinked hydrogels are stable elements as the covalent bonds exist in between the polymer chains. These hydrogels do not dissolve in any solvent until the covalent crosslinked points are weakened. The chemically crosslinked hydrogels are mechanically strong and depend upon the nature of chemical bonds existing between building blocks and crosslinks [18]. Different methods to develop such hydrogels are discussed below:

a) Radical polymerization: Swelling is an important characteristic of the hydrogel system and it can be regulated by the number of crosslinking agents incorporated into the system. Crosslinkers of predetermined properties result in the formation of stimuli-sensitive materials. Natural, synthetic and semi-synthetic polymers are considered to design hydrogels via this route [19].
b) Chemical reaction of complementary groups: The functional groups OH, NH_2_ and COOH present in a polymer make it water soluble and can be modulated to develop a hydrogel. Establishment of covalent bonds between the polymer chains is generally achieved by functional groups and complementary reactivity interaction, such as, amine-carboxylic acid reaction by Schiff base formation.
c) Ionic interaction: Crosslinked polymer systems generated by the inclusion of di or tri-valent counter ions falls in this category. The underlying principle is the gelling of polyelectrolytic solution with oppositely charged multivalent ions. For example, alginate—a polysaccharide with glucuronic and mannuronic acid residues can be crosslinked with calcium ions by ionic interactions at room temperature and physiological pH. Alginate hydrogels are used as templates to encapsulate living cells and release protein. The ionotropic hydrogels tend to degrade under physiological conditions [20].
d) Crystallization: Aqueous solution of water-soluble polymer such as polyvinyl alcohol (PVA) when stored at room temperature forms stable gel. Interestingly, aqueous solution of PVA upon freeze-thawing tends to form a highly elastic and strong gel. The gel characteristics depend on molecular weight of PVA, concentration of the polymer in water, temperature, freezing time and number of freezing cycles [21].

### 2.3. Interpenetrating Network Hydrogel

Based upon preparation method, hydrogels are classified as homo-polymers, co-polymers, semi-interpenetrating networks and interpenetrating networks and are described below:

a) Homo-polymeric hydrogels: Network of polymers derived from single monomer species consist of basic structural units. Homopolymers may possess crosslinked skeletal structure which depends upon monomer nature and polymerization technique. For example, polyethylene glycol (PEG) hydrogels are smart and drug delivery matrices due to their responsive nature to the external stimuli [22].
b) Co-polymeric hydrogels: These hydrogels contain two or more dissimilar monomeric species, among which at least one is a single hydrophobic monomeric constituent assembled in block, randomly or alternating structural configuration along chain of polymeric network.
c) Semi-interpenetrating network (semi-IPN): A linear polymer penetrating into another crosslinked network without chemical bonding is termed as semi-IPN. This network is capable of preserving rapid kinetic response rates with respect to pH or temperature due to lack of restricting interpenetrating network. The hydrogels possess modified pore size and exhibit slow release of drug. The network consists of both covalent and ionic bonds. The covalent bonds maintain three dimensional (3–D) structures of the hydrogels whereas the ionic bond imparts mechanical strength and pH responsive nature [23].
d) Interpenetrating network (IPN): This is an intimate combination of two different kinds of polymers of which one is crosslinked or generated in immediate presence of other polymers. The interlocked structural property of crosslinked IPN system ensures stability and morphology of the hydrogels. These hydrogel templates have appreciable mechanical strength, controlled porosity, physico-chemical and mechanical properties leading to efficient drug loading [24].

### 2.4. Chemical and Stimuli Responsive Hydrogels

These hydrogels are environmentally sensitive and a smart hydrogel system as they experience unusual changes in the network structures, growth factors, permeability and mechanical properties in response to the environmental stimuli. Physical stimuli include light, temperature, pressure, magnetic and electric fields, mechanical stress and chemical stimuli such as pH, chemical agents and ionic factors, that are responsible in changing polymer chain/solvent interactions at the molecular level. The dual responsive hydrogel is a network of two stimuli responsive factors in a single hydrogel system and biochemical stimuli hydrogel responsive to ligand, enzyme and antigen have also shown potentialities in biomaterials field [25].

a) pH responsive hydrogels: These hydrogels with ionic pendant groups response to environmental pH change and have the ability to accept or donate protons. This leads to volume transition to create osmotic swelling force by generating electrostatic repulsive forces in between the ionized groups. pH responsive hydrogels can further be classified as anionic hydrogels with pendant group of carboxylic or sulfonic acid and cationic hydrogels with pendant groups of amines. The degree of swelling of these hydrogels depend on many factors-concentration, crosslinking density, hydrophobicity or hydrophilicity, ionic charge and degree of ionization [25,26].
b) Temperature responsive hydrogels: The change of temperature in the surrounding fluid determines the swelling ability of these hydrogels and can be classified as positive or negative responsive system. Positive temperature hydrogels contract and release solvent from their templates when the temperature is below the upper critical solution temperature (UCST). As the temperature reaches above the UCST, the hydrogel starts swelling. These hydrogels tend to shrink at lower temperature due to complex structure formation by hydrogen bond. At high temperature, the structure dissociates due to hydrogen bond breakage. Similarly, these hydrogels are retrogressive at negative temperature. The thermosensitive hydrogels undergo sol-gel phase transition instead of change in volume at critical solution temperature. Methyl cellulose and hydroxypropyl methylcellulose are excellent example of temperature responsive polymer. Generally, hydrogels formed by IPN are thermo-sensitive and swell at high temperature and shrink at low temperature. Kotono et al. [26] reported polyacrylic acid (PAA) and polyacrylamide (PAA_m_) as positively thermo-sensitive hydrogels. The hydrogel swelling is reversible corresponding to step-by-step temperature change which has effect on release rate of model drug ketoprofen from monolithic device. Overall, the swelling response can be assessed in co-ordination with temperature variation to study the behaviour of hydrogels [27]. The shortcomings of thermo-sensitive hydrogels are non-biodegradability and non-biocompatibility of monomers and crosslinkers which may produce carcinogenic and toxic effects [17]. It is however possible to conceive more biocompatible hydrogels by grafting thermo-responsive polymers, such as poly (*N*-isopropylacrylamide), to polysaccharides [28].
c) Glucose responsive hydrogels: These hydrogels are used for diabetes treatment. They act as glucose sensing carriers in order to trigger insulin release. Such “bio-smart” matrix is engineered by coupling molecular recognition with actuation, which is mainly composed of hydroxyethyl methacrylate (HEMA) and polymethyl acrylate (PMA). Reduction of system’s local pH converts gluconic acid by glucose oxidase in close proximity of oxygen leading to increased swelling of cationic hydrogels, hence release insulin from the system. These gels are promising but exhibit slow response and fail to go back to original conformation while responding to glucose concentrations [29].

### 2.5. Protein Based and Antigen-Responsive Hydrogels

These hydrogels are fabricated with definite sequences, composition and molecular weight by utilizing recombinant DNA technology for biomaterials, tissue engineering and drug delivery purposes. Kopecek and Yang [30] demonstrated crosslinked protein based hydrogel which contains coiled coil triblock co-polymer at the end and water soluble polypeptide domain at the centre. On the other hand, antigen-responsive hydrogel is designed by antigen grafting on hydrophilic polymeric backbone for delivering biomacromolecules at the target site. Miyata et al. [31] reported grafting of antigen with corresponding antibody by introducing crosslinks to the polymer network to develop an antigen-responsive hydrogel. Binding in antigen helps to trigger noticeable change in hydrogel volume. This hydrogel exhibits shape memory characteristics and allow protein permeation through the hydrogel structure with change of antigen concentration [31]. Figure 1 denotes the classification of hydrogels based on the above-mentioned properties.

## 3. Bioengineering Techniques or Approaches that Facilitate Dental Pulp Regeneration

Different authors [32,33] have found that that the cell homing approach, which does not involve the ex vivo culture of autologous cells is the best, most useful and cost-effective strategy for pulp regeneration in dental tissue engineering. Pulp regeneration has become a reality and has been facilitated through molecular and developmental biology or experimental embryology, as well as the biomimetic principles, which mimics the biological processes [34,35,36,37].

Aurrekoetxea et al. [38] stated that cell homing approaches provide an alternative to cell transplantation for dental pulp/dentin regeneration. Tziafas [39] found that regenerative pulp therapy can be used as a bioengineering method or approach that will significantly facilitate pulp regeneration. Regenerative pulp therapy aims at reconstituting the range/continuum of the normal tissues at the pulp–dentine boundary by controlling the tissue-definite processes of tertiary dentinogenesis [35,40,41,42]. In addition, there are several cellular-based tissue engineering approaches that are proven to be appealing and successful thus suitable to replace the traditional surgical and restorative techniques. These latest approaches include the development and use of novel treatments employing several biological compounds along with the cell-based therapies. The approaches are based on the capability of dental pulp and periodontium regeneration through application of molecular biology, stem cell, and material science [38,43].

The use of growth factors and injectable biomaterial scaffolds has accelerated clinical translation and enhanced dental tissue engineering. Based on the scale and purpose of dentin and dental pulp, regenerative endodontics is expected to be the best biological approaches/solutions in regenerative dental medicine [44,45,46]. Additionally, Marí-Beffa et al. [47] stated that endodontics is concerned with the adoption of cell therapy strategies to treat periapical and pulpal diseases. Cell therapy focuses on reconstructing the natural micro-environments, which regulates the activities of dental stem cells [48,49,50].

In vivo approaches can be used to regenerate pulp tissue, thus facilitating pulp regeneration in dental tissue engineering. In vivo approaches encompass the delivery of various allogenic and autologous stem cells, as well as proper scaffolds fused with definite growth factors into root canals to facilitate dental tissue regeneration [51,52,53,54,55]. Regarding the tissue-engineering strategies, active recruitment of endogenous cells (such as progenitor/stem cells) into root canals (cell homing) can be used to regenerate pulp tissues to accelerate clinical pulp tissue regeneration or pulp tissue engineering. Moreover, the use of most common therapy known as the root canal or endodontic treatment is the best approach for dental pulp regeneration [34,35,56,57].

Mao et al., [44] found that endodontic therapies including regenerative endodontic therapy used in dental tissue engineering facilitate effective regeneration of dental pulp. In addition, regenerative endodontic therapy can also be used in primary and secondary canal treatment, and is effective in treating infections or trauma, which involves periapical lesions, dental pulp and dentin [58,59,60,61]. On the other hand, two authors (e.g., [15,62,63]) found that the best strategy that facilitates pulp regeneration involves the amalgamation of disinfections and debridement of infected root canal systems, using and integrating extracellular matrix molecules as well as tissue-definite stem cells.

### 3.1. Potentials Injectable Scaffolds Materials

**Figure 2:**
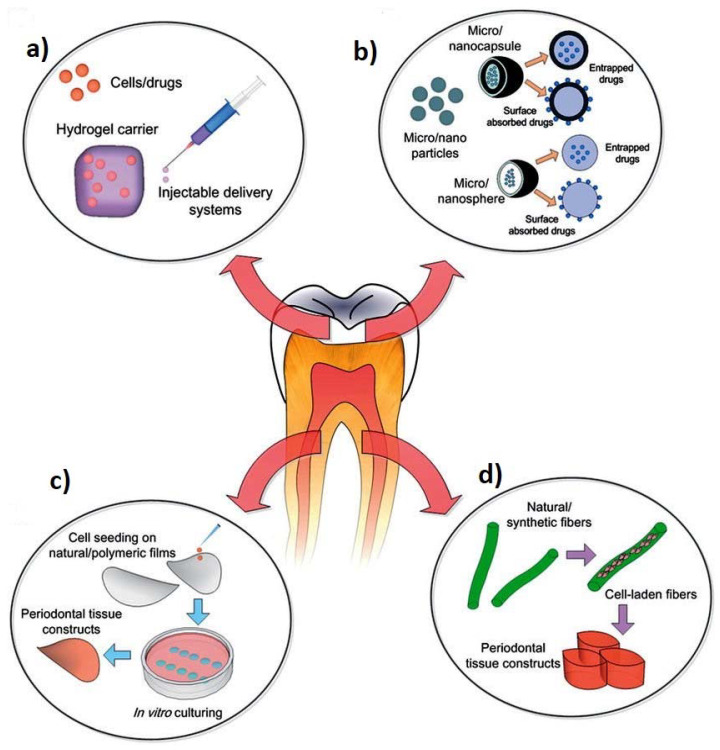
Schematic diagram of different biomaterials as (**a**) injectable hydrogels, (**b**) micro-nanoparticles, (**c**) natural polymers seeded with periodontal ligament (PDL) cells and (**d**) natural/synthetic scaffolds for periodontal disease treatment. Reprinted from [70] with permission from Taylor & Francis Online.

Scaffolds used for tissue reconstruction in dental tissue engineering have significant characteristics such as excellent mechanical and physical properties, vascularization, lower immunogenicity, positive bioactivity and biodegradability without release of toxin or harmful products. They should also demonstrate the ability to remove by-products from the cells easily or effectively, allow diffusion and penetration of nutrients, growth factors as well as cells [45,64,65,66]. Scaffolds used for dental tissue engineering applications have unique distinctions based on their mechanical behaviours. Tissue engineering necessitates rigid scaffolds, which can reproduce the architecture and size of the tissues to be reconstructed/rebuilt [10,14,67]. On the contrary, in the periodontal apparatus of the tissue engineering, as well as in the pulpodentinal complex, because of their smaller sizes and difficulties to get into the receiving sites, the approach presently comprises injectable and soft scaffolds. As a result, the biomaterials utilized to form scaffolds are grouped based on the synthetic and natural sources, or according to the physical steadiness (soft or rigid) [68,69]. Figure 2 represents the schematic diagrams of different biomaterials used for periodontal disease treatment.

Various synthetic polymers are used to create rigid scaffolds such as poly-propylene fumarates, poly-hydroxyalkenoates (PHAs), as well as polyhydroxylacids, whereas majority of natural polymers are utilized mainly in soft matrices [71,72,73]. Apart from scaffold methods, there are other scaffold free methods that are used for dental tissue engineering, which include:

1. Conventional method: This method includes root canal disinfection via irrigation, intra-canal dressing application by using antibiotic pastes, introducing mechanical irritation of remaining pulp/periapical tissues to provoke bleeding into the root and mineral trioxide aggregation (MTA) placement on to the blood clot.
2. Classical tissue engineering approach:
  a) Periodontal regeneration: such as the cell sheet technology that includes a non-invasive method, which uses a thermos-responsive polymeric material for example, poly *N*-isopropyacrylamide (PIPAAm) [74].
  b) Pulp reconstruction: Regarding pulp regeneration, the scaffold free approach involves: (i) endogenous stem cells from resident dental pulp/periapical tissue, growth factors and chemo-attractants to induce migration, proliferation and differentiation of cells and (iii) injectable bioactive scaffolds to initiate cell mediated degradation that is replaced by natural extracellular matrix (ECM) [75].

### 3.2. Periodontitis and Treatment Objectives

Periodontitis, a common disease in the dental field is an inflammatory response initiated by plaque micro-organisms. Periodontitis involves a cascade of inflammatory process which progressively destroys tooth supporting tissues such as gingival, periodontal ligament, alveolar bone and root cementum [76].Clinical explanation of periodontitis defines the proportion of the site which bleeds on probing and number of proportion of teeth with depth of probing (>4 and >6 mm) and of teeth (>3 and >5 mm). Biofilm consisting of micro-organism community remains embedded on tooth surface and gingival sulcus. To maintain appropriate dental health, biofilm accumulation and aggregation need to be discouraged. This is primarily accomplished by using dentifrice, such as compounds including abrasives, detergents and antimicrobials, along with mechanical tooth brushing. Untreated periodontitis causes increased mobility of tooth, loss of tooth and may also lead to irreversible destruction of periodontium. All these factors hinder normal mastication, food intake and aesthetics [77].

Periodontal health is necessary for successful comprehensive dentistry. In order to achieve long time therapeutic comforts and predictable treatment, active periodontal infection need to be controlled before initiating restoration of implant dentistry [78]. The proper treatment plan concerned with the establishment and maintenance of oral health includes need of emergency pain and acute infection treatment as

a) Removal of affected teeth;
b) Non-surgical followed by surgical periodontal pocket therapy techniques;
c) Endodontic therapy and occlusion correction including orthodontic therapy;
d) Need of prosthetic replacement for teeth abutments in case of fixed prosthesis;
e) Aesthetic consideration in periodontal therapy;
f) Therapy sequences.

**Figure 3:**
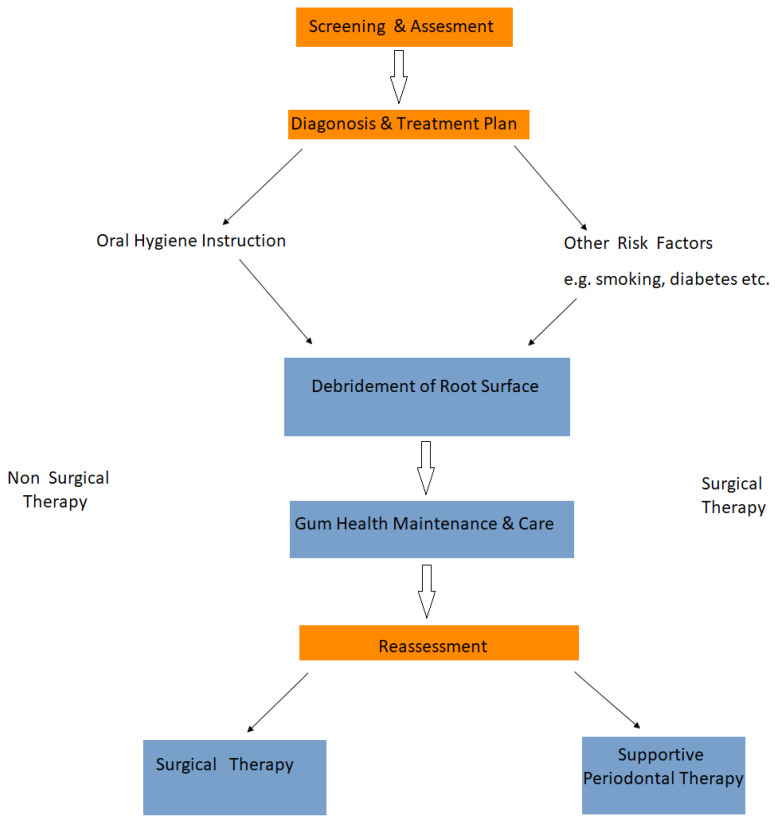
Representation of distinct stages of periodontal assessment. The examining and monitoring part including assessment and diagnosis/ treatment plant are denoted in orange whereas the surgical and non-surgical parts included in maintenance and cares are denoted in blue.

The distinct stages periodontal assessment and treatment is demonstrated in Figure 3.

Based upon these treatment plans, several models were developed for the purpose of periodontal treatment [79]. Among different models, the two most established models are briefly discussed below:

Model 1: Periodontal treatment planning: The treatment undergoes in four distinct phases: systemic therapy phase of smoking, counselling, cause related therapy-hygiene phase of periodontal therapy, cause related therapy with corrective phase i.e., measure to take up periodontal surgery, endodontic therapy and maintenance/care therapy.

Model 2: Periodontal treatment planning: Here, the treatment is divided into following phases:

(i) Preliminary phase: (a) Emergency purpose: dental/periapical and periodontal and (b) hopeless teeth extraction followed by provisional replacement.
(ii) Non-surgical phase: (a) Plaque control and patient awareness/education, (b) removal and planning of root followed by correction of prosthetic irritation factors, (c) excavation and restoration of caries, (d) antimicrobial treatment and occlusion therapy and (iv) minute orthodontic movement and provisional splinting.
(iii) Response to non-surgical phase: Pocket depth-gingival information and plaque, calculus and caries investigation.
(iv) Surgical Phase: Periodontal therapy with implant placement and endodontic treatment.
(v) Restoration period: (a) Final restoration with fixed and removable prosthodontic appliances and (b) response to restorative process.

Apart from all these factors, some of the important sequences involved are:

(i) Emergency treatment which include root canal extraction to treat abscessed teeth and treatment of periodontal defects.
(ii) Antimicrobial therapy which is utilized mostly at local site in periodontics. This includes regular mouth wash and delivery of antimicrobials locally into the periodontal pockets.
(iii) Diet control is also an essential means for periodontitis preventing program. Deficiency of iron, zinc, vitamin B12, C or D needs to be addressed from the start of periodontal treatment. Patients suffering from diabetes should undergo medication and diet therapy to make them aware of periodontal treatments.
(iv) Patient education and motivation: Patients should understand the treatment plans initiated by the doctors to learn and practice oral hygiene measures for proper maintenance of teeth [79].

If the periodontitis is not advanced, invasive procedure is considered as the primary treatment mode as:

a) Scaling: It eliminates bacteria and tartar from the surface of the tooth and beneath the gums with the help of instruments, ultrasonic device or laser.
b) Root planning: It helps in smoothening of the root surface and does not permit further growth of bacteria and tartar. It prevents inflammation and fastens gum re-attachment to the tooth surface.
c) Antibiotics: Oral as well as tropical antibiotics prevents the spread of bacterial infection. This includes mouthwash followed by gel insertion containing antibiotics to be applied in between the spaces of the gums and teeth. Such gels can also be applied into the pockets after deep cleaning.

With advanced periodontitis, the gum tissue is pulled away from teeth allowing additional bacteria to be built up at the affected site and cause severe infection. The treatment of advanced periodontitis involves:

a) Flap pocket reduction surgery: It involves cleaning of periodontal pockets to allow exposure of the root for better scaling and root planning. In this case, the underlying bone can be re-contoured before suturing of gum tissue.
b) Soft tissue grafts: These include the removal of small portions of tissue from the palate by utilizing donor tissue and constructing it on to the affected site. This prevents gum recession and covers up the exposed roots.
c) Bone grafting: This procedure is taken into consideration when periodontitis is featured with destruction of tooth root by the surrounding bone. Bone grafts serve as a platform for bone re-growth [80].

Various tissue regenerative approaches such as the use of bone grafts along with growth factors and occlusion barrier membranes have been proposed to restore lost tooth supporting tissue. Some of these approaches achieved partial success in repairing and regenerating damaged periodontal tissue, but the outcomes are still unpredictable. Even, the gold standard bone grafts have a failure rate of 30% in maxillofacial and craniofacial surgeries apart from limited availability and donor site morbidity. Periodontal tissue has the ability to regenerate itself, thus, substantial efforts in the tissue engineering field have been carried out to overcome limitations arising with periodontitis treatment.

#### Bioactive Scaffolds: Hydrogels for Periodontal Regeneration

Hydrogels or more specifically injectable hydrogels can serve as potential biomaterials for pulp and periodontal regeneration. Self-assembling peptides are often modulated into nanofibers due to electrostatic and hydrophilic-hydrophobic interactions. Such self-assembled peptides trap water and form gels. The peptide enriched template based on matrix-metalloproteinase 2-sensitive enzyme cleavage site and tripeptide arginine-lycin-aspartic acid (cell adhesion motif) along with growth factors bind by heparin enacts as suitable hydrogel system for periodontitis regeneration. Such growth factors include GF β1, vascular endothelial growth factor (VEGF) and basic fibroblast growth factor 2 (bFGF 2).

**Figure 4:**
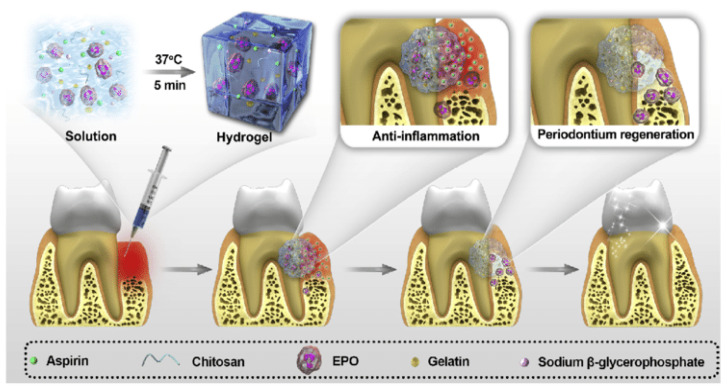
Schematic representation of thermoresponsive hydrogel prepared from chitosan/sodium-β-glycerophosphate/gelatine for promoting periodontal regeneration. Reprinted from [81] with permission from Elsevier.

Xu et al. [81] designed an injectable and thermosensitive hydrogel made of chitosan/ β sodium glycerophosphate (β-GP)/ gelatine blend to terminate alveolar bone resorption, anti-inflammation and regeneration of periodontitis and is depicted in Figure 4. The sol-gel transition of the blend occurred at body temperature. These hydrogels have the ability to release aspirin and erythropoietin to induce anti-inflammatory effects and guide tissue regeneration. The releasing profile suggested that the released materials were non-toxic both in vitro and in vivo, thus demonstrating potentialities of the hydrogel system in periodontits treatment applications. Yan et al. [82] suggested the preparation and in vivo evaluation of enzymatically solidified chitosan hydrogel to treat intrabony periodontal defects. These hydrogels are highly biocompatible and biodegrable. Tan et al. [83] used hydrogelator Na_p_FFY to combine with SDF-1 and BMP 2 to fabricate supramolecular hydrogel. The bioactive factors (VEGF, platelets derived growth factors) released from hydrogels promoted reconstruction of periodontal tissue in rat model by affording sufficient and continuous stimuli. These hydrogels could replace bone transplantation in the clinic in future to repair periodontal bone defects and periodontically accelerated osteogenic orthodontics. Moreover the combination of bioactive glass nanoparticles in these thermosensitive hydrogels could be also a possibility to enhance the osteoconductive of such biomaterial.

### 3.3. Natural Polymers as Scaffold Materials

#### 3.3.1. Fibrin as Scaffold Materials

Fibrin gel is prepared by the reaction between commercially purified allogenic fibrinogen and thrombin which are the key proteins involved in blood clotting [84]. Fibrinogen is basically a rod-shaped protein which has three pairs of polypeptide chains connected by disulphide bonds. It plays a major role in haemostasis, platelets attachment and aggregation. Fibrinogen to fibrin gel transformation includes different distinct steps: (i) thrombin cleaves out fibrinogen to tiny fibrino peptides α_2_, β_2_ and γ_2_, (ii) thrombin solution in presence of calcium ions stimulates Factor XIII to form transglutaminase-Factor XIIIa and (iii) Factor XIIIa catalysation crosslinks fibrinopeptides to transform to fibrin network which is the hydrogel [85]. Fibrin crosslinks also play an important role as the crosslinking of fibrin clots by Factor XIIIa increases the stability of clot via increased clot stiffness and deformation resistance by decreasing susceptibility to degradation. Factor XIII is an inactive precursor which circulates in plasma and activates fibrinogen by proteolytic cleavage using thrombin, thus, denoted as XIIIa. Fibrinopeptide release plays a huge role in maintaining overall fibrin structure than XIIIa crosslinking [86].

Fibrin is used to develop biological polymers scaffolds used in the tissue bioengineering. Fibrin on its own or the combination of fibrin with other materials offers very efficient scaffold materials that can apply to the stem cells or the primary cells to induce regeneration of adipose tissues, cardiac tissues, liver, ocular, nervous tissues, skin, bones, cartilages, tendons, and ligaments. Hence, fibrin is one of the most versatile bioengineering materials with a vast potential of application to facilitate dental regeneration, wound healing and tissue regeneration. There are various forms of prepared fibrins. For instance, autologous plasma may be useful in developing the fibrin. The autologous plasma is a readily available material in the form of engineered microbeads and glue [87,88]. Flanagan et al. [89] found that using fibrin as a cell carrier material in various tissue engineering applications leads to autologous and mechanically stable structures, which undergoes noteworthy tissue development in vivo. Fibrin can also be used as an autologous scaffold biomaterial to develop implantable structures that can be used in dental engineering or dentistry.

Commercially available fibrin gel product is mainly designed for the purpose of tissue/organ regeneration. Thus, thrombin of higher concentration (approximately 10–100 U/mL is used to prepare gelation as short as possible by considering the clotting time. A comparatively longer time of gelation and adequate strength are necessary for the fibrin gel to be utilized as an injectable scaffold. Thus, injectable properties, handling convenience, cell entrapment and cell growth performance are considered to optimize gelation condition [90]. Zhao et al. [85] optimized the fabrication and gelation condition of fibrin gel to develop commercially available hydrogel for cell entrapment and delivery. It is reported that the fibrin glue polymerization fastens with the increase of thrombin concentration. According to gelation mechanism, fibrinopeptide concentration and fibrin gel monomer play vital roles in the clotting time. With the increase of concentration of thrombin, there is the production of more fibrinopeptides per unit by the fibrinogen cleavage, leading to shorter clotting time. The thrombin is greatly influenced by temperature as fibrinopeptide formation per unit is high at higher temperature. Additionally, the fibrin gel structure is strongly influenced by the gelation kinetics i.e., the time of clotting. Thus, as the thrombin concentration increases, the gelation time fastens, resulting in more fibrous structure of fibrinogen hydrogels. The stability of hydrogel is also influenced by the gelation. The crosslinked fibrin gel has the ability to resist thermal disintegration after gelation. The crosslinking structure of hydrogels depends upon thrombin concentration. With higher concentration of thrombin, the crosslinked fibrin gelexhibits more organized and complete crosslinking structure and enables stronger ability to resist shrinkage, leading to high water uptake. This water uptake property of hydrogel favors free exchange of nutrients for hydrogel system for cell delivery purpose [14,91].

Kretlow et al. [92] conducted a study to establish the possible biomaterials that can be injected to achieve the regeneration of complex tissues. Injectable biomaterials, especially those gathered from aqueous solutions serve as excellent carriers for cells and other bioactive elements can be used for dental tissue engineering. Rajangam and An [93] found that fibrin (Fbn) and fibrinogen (Fbg) can be used as scaffolds because they tend to achieve uniform cell distribution, a higher cell seeding efficiency and uniform cell distribution, as well as it has the ability to migrate, proliferate, and differentiate into definite organs/tissues by secreting ECM (extracellular matrix)to generate tissues. Therefore, fibrin is an ideal material as a scaffold biomaterial for dental tissue engineering applications. Fibrin (Fbn) and fibrinogen (Fbg) has been developed into different scaffolds due to their nano and micro characteristics. Various scaffolds including nanoporous or microporous scaffolds i.e., hydrogels, [94], nanofibers [95], nanoparticles [96], microfibers [97], microtubes [98], and microspheres [99] have been formulated for different tissue engineering uses such as dental tissue engineering. Fibrin and fibrinogen scaffoldsplay an important role in the initial phases of wound repair and hemostasis, as well as blood clotting. They are also natural provisional extracellular matrix. Fibrinogen has a higher affinity towards biomolecules, for example, tissue-inductive cytokines, fibroblast growth factors (FGFs), as well as vascular endothelial growth factors (VEGFs); therefore, it can be used as injectable biomaterial scaffold for dental tissue engineering [6,100,101]. Fibrinogen offers a favourable surface for cellular proliferation and attachment comprising a 3D fibrous structural support as well as nano-textured surfaces that includes a fibrous network for cell to cell interaction, cell matrix or cell signalling [102]. On the other hand, fibrin gel is used as an injectable biodegradable scaffold in dental tissue engineering because it offers numerous benefits, such as, it degrades in controllable way, supports better cell attachment, and exhibits great biocompatibility features [103]. Particularly, in dental or other tissue engineering, fibrin and fibrinogen-based scaffolds induce formation of extracellular matrix production, which provides support to connective tissues, for example, skin, blood vessels, nerves, tendons, bones, ligaments and cartilage [93,100,104,105].

#### 3.3.2. Use of Chitosan as the Scaffold Material

Chitosan, a natural biomaterial is purified from chitin whose major source remains mostly crustacean’s (crab and shrimp) exoskeleton. Chitosan consists of *N*-acetyllucosamine and glucosamine co-polymer units. This polymer is antibacterial, biocompatible and is generally combined with different bioactive matrices to induce osteoconductive properties into them. These unique properties of chitosan have led to exploit it particularly in bioengineering and tissue engineering field and also in regenerative medicine [106].

Chitosan can be tailored in different formats as gels, scaffolds, fibres etc. Chitosan’s properties depend mostly on its molecular weight and its degree of acetylation. The extraction source and procedure involved in acetylation determine the polymer’s final properties. The deacetylation strength influences the physico-chemical and biological properties of the polymer as well. At low pH, the pH versatility of this polymer results in amine groups protonation, thus, exhibiting a polycationic nature. Chitosan amine tends to deprotonate at higher pH, hence, promotes intermolecular interactions to form film, fibres, scaffolds and gels. Various chemical modifications along with crosslinking can improve mechanical, biodegradable properties and cell affinity of chitosan. This polymer also acts as an excellent antimicrobial agent and exhibits high degree of biocompatibility in the animal model, hence, can be adapted conveniently to develop implantable biomaterials. Chitosan is considered as an emerging and potential biomaterial for dental application due to its antimicrobial, biocompatible properties, bioactivity and ability to blend with other materials [107].

Chitosan based composites (CBC_s_) are used to tailor local drug delivery system with appropriate contact time, mechanical characteristics and sustained drug release profile, while keeping intimate contact with the oral mucosa. Chitosan microspheres have been fabricated to initiate active release of drug at pathological sites [108]. This polymer has been widely explored for drug delivery for DNA, small interfering RNA (siRNA), growth factors and different drugs. Oral administration of chitosan is found to be non-toxic in nature [109]. Chitosan as nano-particles and films help to deliver antibiotics such as metronidazole and nystatin to treat periodontal tissues in situ with fungal infection and oral mucositis. Thiolated based chitosan formulation is entrapped in muco-adhesive patches to cure dental caries by sustained antibacterial medicament release, thus, inhibiting cariogenic *Streptococcus mutans* growth [110], and these systems offers also the advantage of being pH-sensitive. Hydroxyapatite (HA)/ chitosan blend acts as antibacterial medicaments and dentifrices for oral environment. Chitosan has also exhibited effective plaque control element in vitro by causing inhibition of particular dental plaque pathogens such as *Actinobacillus actinemycetecomitans*, *S. Mutans* and *P. Gingivalis* [111].

Chitosan is also considered as a favourable matrix for periodontal tissue regeneration. Few investigations accounted for the development of chitosan membranes impregnated with bioactive material-HA, calcium phosphate variants such as tricalcium phosphate (TCP) α and β to treat periodontitis. Also bioactive glass nanoparticles have been combined with chitosan to produce composite membranes for periodontal regenerationThe characteristic features of these blends are also improved by adding crosslinkers such as glutaraldehyde and genipin into the blend to improve mechanical properties (elastic modulus, toughness and hardness) of the composite chitosan blend membranes. Chitosan/HA has been extensively investigated by Oliveira et al. [112] as layer by layer chitosan/HA composite materials using rapid prototyping system. Chavanne et al. [113] worked on similar pattern and fabricated porous cylindrical templates for the treatment of periodontitis. Qasim et al. [114] has utilized freeze gelation technique to fabricate core layer boosting material as mechanically strong, biocompatible and porous chitosan/HA membranes using ascorbic and acetic acid as solvents. A bioceramic layer of unique crystallinity was detected on the matrix with a chitosan backbone. Such a graded condition is required for tissue implant interface.

Chitosan has also been explored as dentifrices for dental tissue engineering purposes. Ganss et al. [115] has reported commercially available chitosan dentifrices which is non-fluoride in nature and reduce loss of tissue. Such dentifrices hinder erosion of dental matrix and enamel which is attributed to cationic nature of this polymer coupled with low pH. For human enamel regeneration, chitosan-based formulations have been taken into account by imparting organic amelogenin delivery at the enamel defect sites. Ruan et al. [116] used chitosan hydrogel to transport amelogenin to rejuvenate aligned crystal structure. The polymer generates a protective effect against the secondary caries with respect to antibacterial characteristics and does not influence orientation of enamel crystal. For adhesion and dental binding, antioxidant chitosan hydrogels with β-carotene and nystatin were investigated to validate delivery of robust dentine bonding system with improved mechanical strength (sheer bond of 38 MPa after 24 h and 20 MPa after 6 months).

Biomimetic dental restorative materials are widely investigated nowadays for clinical application. The most common material used for this purpose is glass ionomer cements (GIC_s_) (fluoroaluminosilicate glass powder with PAA liquid) which forms a chemical attachment with the calcified tooth tissue. GIC_s_ have favourable physico-chemical properties, antibacterial effects, biocompatibility, sustained fluoride release and high affinity for tooth structure (enamel dentine). But GIC_s_ are associated with poor fracture toughness and insufficient bulk-filled restoration. Thus, chitosan is often combined with GIC_s_ to improve the mechanical properties of the cement [117]. Petri et al. [118] reported enhanced value of flexural strength of cement post blending of chitosan polymer which has also increased fluoride ions leaching rate from the set materials. These blends have immense potential in the field of bioactive dental restorations as well as regenerative endodontics especially in case of pulp therapy.

Chitosan is also explored widely as coating dental implants. The polymer coating has positive effect on the surface and bone interface by alteration, morphological, mechanical and biological factors. For example, chitosan coating tends to change elastic modulus, thus minimizing mismatch between the alveolar bone and implant surface by reducing the stress concentration area. Additionally, such coating potentially provides various medicaments, such as antibiotics for local drug delivery around the implant area [119].

Stem cell transplantation strategy has an immense potential in the dentistry field and can alleviate oral conditions by treating periodontal diseases using embryonic stem cell (ESCs) and adult dental stem cells to induce pluripotent stem cells (iPSCs) in regenerating tooth. Researchers have found that chitosan acts as an important carrier for chitosan-mediated stem cell repair [120]. Yang et al. [121] reported utilisation of dental pulp stem cells grown effectively on collagen-chitosan blend system in order to form dentine-pulp complex. Chitosan, thus, is considered as one of the most promising biomaterials for dental applications which range from restorative dentistry to engineered dental scaffolds to periodontal complex healing. Very little clinical data is available on dental clinical applications for this specific polymer. There is further necessity of research, especially in in vivo studies as well as clinical trials for the chitosan-based membranes [107].

#### 3.3.3. Use of Alginate as the Scaffold Material

Alginate is an anionic polysaccharide or specifically salt of alginic acid and is usually isolated from brown algae. Alginate is composed of (1–4) linked β-D mannuronate and C-5 epimer α-L-guluronate residues and can be crosslinked with divalent metal ions to produce water soluble hydrogels which permits their immense use in the dental field [122].

Diniz et al. [123] developed an injectable and biodegradable scaffold made of oxidised alginate microbeads encapsulated with periodontal ligament as well as gingival mesenchymal stem cells. The stem cells were incorporated into the alginate hydrogel in vitro. The scaffold system exhibited high swelling ability and osteo-differentiation and adipo-differentiation. There was a formation of an HA-like crystalline structure in the microbeads which confirmed the potential application of the scaffold for dental application. Devillard et al. [124] fabricated collagen/alginate based stem cell composite scaffolds for the purpose of endodontics regeneration. One of the major issues regarding inflammation in the dental implantology is peri-implantitis. To solve this purpose, silver lactate incorporated RGD-coupled alginate hydrogel system with stem cells was tailored with excellent antimicrobial properties against *Aggregatibacter actinomycetemcomitans* [123].

Alginates are easy to use and well tolerated by patients, thus, it can be used extensively for primary prosthetics, orthodontics and design imprints. In dentistry, alginates are prepared as powder which when mixed with water form soft paste and delivered readily into the oral cavity for impression detection. The alginates available in the market for dental impression are of two types: fast setting (hardening time of 1–2 min) and normal setting (setting time of 2–5 min). These impression materials are easy to use and have favourable setting time [125]. Sancilio et al. [126] demonstrated fabrication of alginate/HA based nanocomposite scaffolds for possible dental pulp biomineralization and differentiation. HA encourages inorganic reinforcement with alginates and the osteoconductive component of the blend system. Generally, human dental pulp stem cells (DPSCs) are capable of differentiating in multiple lineages. The authors have aimed to verify mineralization and differential potentialities of human DPSCs seeded on to the synthesized scaffolds. DPSCs expressed osteogenic differentiation related markers and promoted deposition of calcium as well as biomineralization onto alginate/HA scaffold matrices. Dobie et al. [127] investigated utilization of alginate hydrogels to present TGF β1 to dentin pulp complex to signal reparative processes. Alginate hydrogels were applied to cultured human and initiate upregulated dentin matrix secretion. The hydrogels also initiated odontoblast-like cell differentiation on cut pulpal surfaces. Alginate hydrogels provided a suitable template to support dental regeneration and to deliver growth factors to enhance natural regenerative ability of the dental pulp.

#### 3.3.4. DNA/Protamine Complex as the Paste for Injectable Material for Dental Applications

Fukushima et al. [128] used a compound powder of DNA/protamine that was developed through the reaction of DNA with a solution of protamine sulphate to develop injectable material for dental applications. The mixture was stirred effectively to achieve new injectable material to use in dentistry treatments. The powder generated was put under the kneading process to form a paste. The complex was tested using the FT-IR technique. The result of the FT-IR measure revealed that the complex formed had a viscosity of about 280.1 pas and a relatively porous structure [128,129]. As a result of a needle 0.25 mm diameter easily passed through the paste. Therefore, the result confirmed that DNA/Protamine paste had the desired viscosity to apply in the clinical field as a perfect injectable biomaterial. In addition, the paste complex revealed to be capable of delaying the growth rates of *Staphylococcus aureus* and the *Porphyromonas gingivalis* for a short time. However, the paste complex was unable to kill or inhibit the growth and multiplication of bacteria [128,129]. The disk from the paste indicated the mild response of the tissue that later degraded because of implantation to the soft tissues. As such the result suggests that the DNA/protamine paste is an excellent biodegradable material. Thus, the material is recommended for use as an injectable scaffold through using syringes to repair defected tissues, guided tissue regeneration (GTR) and guided bone regeneration (GBR) dentistry treatments [128,130].

#### 3.3.5. Chitosan-Hyaluronic Blend Hydrogel

Miranda et al. [107] investigated about hydrogel acquired from chitosan-hyaluronic and its applications in periodontal tissue engineering. The freeze-drying technique was used to collect the chitosan and hyaluronic acid hybrid. Miranda and others argue that developing periodontium for treating periodontitis is a very crucial challenge. In the study, a modified hyaluronic acid together with chitosan developed the chitosan-hyaluronic acid hybrid (CS-HA) hybrid. The CS-HA hybrid generated a perfect scaffold material for periodontal tissue regeneration. The study concluded that hydrogel acquired from chitosan-hyaluronic can be used to create scaffolds that is used to support osteoblasts, gingival, cementoblasts and support the periodontal fibroblast cells, as well as used for periodontal tissue engineering [107].

The chitosan-hyaluronic acid hybrid hydrogel can be used as scaffold material for dental tissue engineering. The chitosan-hyaluronic acid hybrid hydrogel can also be used to address osteoblasts, gingival, cementoblasts and supports the periodontal fibroblast cells [131]. Natural polymers are utilized in synthesis of injectable hydrogel for tissue regeneration such as in dental tissue engineering. The use of the chitosan–hyaluronic acid (CS/HA) hydrogel in periodontal tissue engineering prevents any complications and promotes effective dental tissue regeneration [132]. Hydrogel acquired from chitosan–hyaluronic and its applications in periodontal tissue engineering and hyaluronic acid-based scaffolds is used in controlling cell response and constructing ultimate tissue engineering products, for example, in dental tissue or other tissue engineering applications [133]. On the other hand, hyaluronic acid is a commonly used natural materials used to fabricate scaffolds as hydrogels [134,135]. The hydrogel scaffolds are used to regulate inflammation responses, cell angiogenesis, migration, as well as differentiation of cells, and thus they are ideal scaffold materials for tissue engineering due to its non-toxic degradation and excellent biocompatibility features [136,137].

### 3.4. Synthetic Polymer as Scaffold Material

#### 3.4.1. Emdogain and Its Use in Dental Field

**Figure 5:**
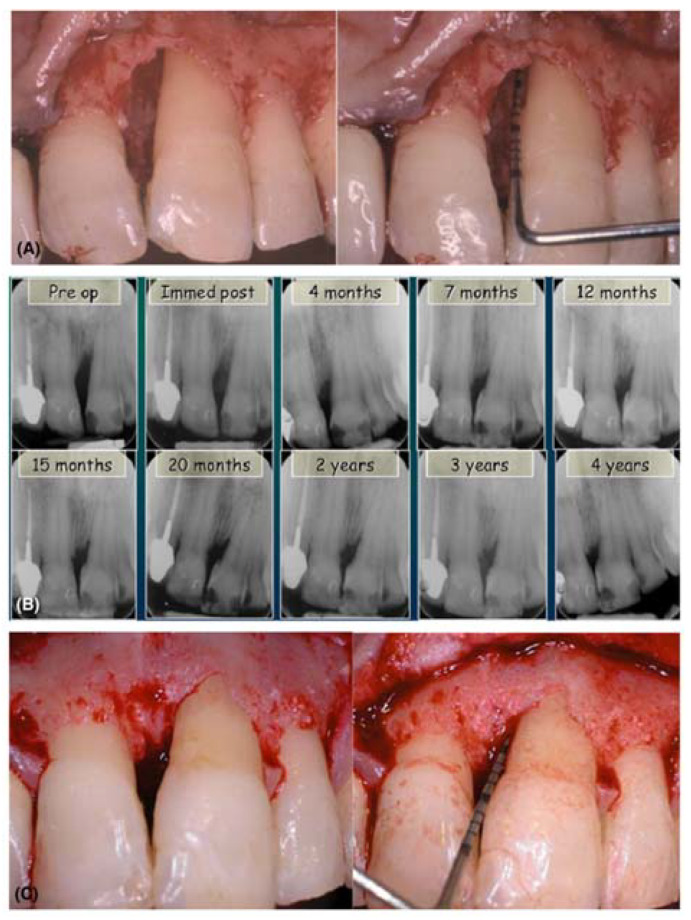
(**A**) Pre-operative clinical images showing a deep intrabony defect, (**B**) X-rays depicting appreciable defect fill following treatment with EMD in combination with a bone graft, and (**C**) four year outcome following intrabony defect repairment with EMD. Reprinted from [140] with permission from Wiley Online.

Enamel matrix derivative (EMD) is a commercially available biomaterials which is mainly used for the treatment of intrabony periodontal defects and to restore soft tissues. The protein EMD is extracted from unerupted porcine tooth buds and it contains amelogenin (90%) and small amount of ameloblastin, enamelin, tuftelin and few other nanomelogenin proteins. The major application of EMD is to stimulate newly periodontal attachment formation for acellular cementum, periodontal ligament (PDL) and alveolar bone. EMD also has the ability to induce regeneration of the periodontal tissue. It is the most widely studied biomaterials in dentistry and has also been extensively used in surgical procedures such as root end resection with guided tooth tissue regeneration [138]. Besides, EMD is applicable in various disciplines of dentistry such as implantology, traumatology, endodontics as well as wound care. The versatility of EMD lies in its application to treat and promote dentin tissue formation by regulating dentin sialoprotein, TGF-β, collagen and bone morphogenetic protein (BMP) expression. EMD or the commercially named Emdogain is supplied in sterile aqueous propylene glycol alginate (PGA) solution. This PGA solution facilitates Emdogain application on to root surfaces which are exposed during periodontal surgery [139]. Figure 4 shows the effect of emdogain in combination of bone graft to treat intrabony defects. Figure 5 represents the clinical image of a deep intrabony defect and its subsequent healing (X-ray images) after treatment with EMD along with a bone graft.

Heijil et al. [141] demonstrated that the tropical application of Emdogain on defected root surfaces associated with the intrabony defects during modified widman flap (MWF) surgery promoted improved gain of radiographic bone as well as clinical attachment compared to placebo surgery in the same patient. On the other hand, few researchers have pointed out the concerns regarding viscous nature of EMD which may not prevent flap collapse. EMD along with grafting material was tested to overcome this potential limitation [140]. Al Hezaimi et al. [142] found that EMD is more efficient in case of reparative dentin formation as compared to calcium hydroxide [Ca(OH)_2_] and takes direct part in pulp capping procedure. One of the most important and promising application of EMD is to promote regeneration of functional PDL after avulsed teeth avulsion and replantation [143].

In terms of revascularization, Nakamura et al. [144] utilized EMD as intracanal medication in animal (rat) model to find out its efficiency in inducing apical periodontitis treatment. The teeth treated with EMD exhibited a rising level of TGF-β1, BMP-2 and cementum regeneration around root apex after two weeks when compare to control animals. EMD has the capability to modulate osteogenic environment by minimising RANKI and by increasing production of osteoblastic osteoprotegerin. EMD has positive influence on cementoblast activity and is found to be one of the best materials for inducing human tooth germ stem cell proliferation and differentiation as compared to Ca(OH)_2_ and MTA. Besides, EMD has been successfully used to treat large periradicular lesions of endodontic origin located adjacently to dental implants with regressive peri-implantitis [145]. Sarnast et al. [146] found that regressive peri-implantitis (basically, an endodontic infection from neighbouring teeth) can be treated by initiating infected tooth apicoectomy with dental implant decontamination. This treatment can be performed by EMD application which aids in periapical lesion healing.

Palioto et al. [147] evaluated the attachment, proliferation, differentiation and expression of collagen Type Iin PDL fibroblasts that are stimulated by EMD/ insulin-like growth factor 1(IGF-1) in a time dependant manner. Keila et al. [148] investigated that in rat model, osteogenic capacity of bone marrow and mineralized nodule formation are enhanced by EMD. The growth, adherence and metabolic activity of PDL significantly increased with the addition of EMD which resulted in the conversion of pluripotent immortalized mouse myoblast cell line (C2C12) mesenchymal cells differentiation pathway into osteoblast lineage.

Schwarz et al. [149] investigated EMD effect on human osteosarcoma cell line (Saos-2) osteoblast proliferation, differentiation and viability on titanium implants. EMD was found to enhance SaSo2 osteoblast viability and stimulated fibroblast growth factor 2 expression but simultaneously minimised alkaline phosphatase expression.

#### 3.4.2. Pegylated Gel of Fibrin

PEG is suitable for providing a temporary matrix [150]. Galler et al. [14] examined the use of PEGylated gel of fibrin in the bioengineering of stem cells of the dental system, and found that stem cells acquired from the postnatal systems can offer useful structures for the specific part of a tooth after an in vivo transplantation. On the other hand, stem cells acquired from the postnatal systems can offer useful structures for the specific part of a tooth after an in vivo transplantation. Scaffolds that are compatible with stem cells of the dental systems can also be used in tissue engineering in dentistry [151,152]. Honda et al. [153] reviewed the use and performance of adult dental cells and postnatal cells in dental tissue or tooth regeneration, and found that postnatal cells can be applied in the tissue engineering to regenerate dental systems, which acts as a great innovative approach. The authors also proposed the use of biological strategies to reaerate dental or tooth root using postnatal dental cells. PEGylated fibrin material is suitable material that can be used in the dental stem cells. Fibrin is a bipolar material that plays very crucial roles in the wound healing process and homeostasis. However, one of the key advantages of fibrin is that it degrades easily. Thus, to address the issue of degradation, fibrin is modified with “polyethylene glycol” (PEG), resulting in the formation of a hybrid material that facilitates the cell delivery in dental tissue engineering [154].In their study, Galler et al. [14] combined the PEGylated hydrogel of fibrin with the stem cells acquired from the periodontal ligament or the pulp. The in vivo study of transplantation provided vascularized connective tissue that simulates the dental pulp. Their findings show that that fibrin facilitates growth and development of stem cells through differentiation, and thus can be used in dental tissue engineering to facilitate dental pulp regeneration. Moreover, the use of vascularized connective tissue, which simulates the dental pulp and fibrin facilitates growth as well as development of stem cells through differentiation, and the use of PEGylated materials will also facilitate the regeneration of dental pulp, dentin and tooth root [155].

#### 3.4.3. Suitability of the Synthetic and Natural Biomaterials (Poly-Lactic Acid and Collagen)

Studies [156,157,158,159] found that various synthetic and natural biomaterials are also suitable for application in dental tissue engineering, especially for the pulp. Moreover, injectable biomaterials, stem cells, poly (lactic acid)-based biomaterials, collagen and hydrogen are also suitable for dental tissue engineering. Tissue engineering that focuses on the dental pulp possibly succeeds after insertion of the stem cells gathered from the pulp and the combined with the scaffold materials that are inserted inside the emptied root canal. The common materials applied for this treatment are the collagen also known as poly (lactic) acid [157].

However, the use of these materials faces the challenge due to difficulties in insertion or in the modification to fit into narrow spaces of the pulp. As a result, there is the need for other materials which are simple to insert and to modify [45,160]. Currently, there are new hydrogel materials that contain some bioactive component have been proven effective because one can modify the elements to fit specified use thus establish an optimized result [161].

In their study, Galler et al., [156] tested different natural hydrogels alongside other synthetic materials which were tested in order to find the most suitable material to steer the engineering of the dental pulp. The study used two forms of polyethylene glycol and compared the contents to fibrin, collagen, and the self-assembling peptide. Galler et al. [156] assessed the viability of the stem cells used in developing the test materials after two weeks. Further, the study used the specific cells landed with growth factors acquired from the dentin got inserted to a root of the human tooth, which was implanted subcutaneously to mice that are immune compromised [156]. The study finding revealed that the process of in vitro culturing of the cell represented a significant difference in relation to the type of scaffolds. It also indicated that viability in natural material was higher than that of the synthetic materials. On the other hand, the in vivo experiments revealed significant differences in terms of the scaffold’s rate of degradation, the formation of soft tissues, vascularization, and the type of differentiation that resemble the odontoblasts cell differentiation [156]. Various studies [45,67,156,162,163] found that fibrin is the most suitable material because it enhanced the generation of the pulp resembling tissues and facilitated the odontoblasts cell differentiation at the cell dentin interface. The use of fibrin is the most suitable naturally occurring material because it has superior abilities compared to the synthetic scaffolds in terms of viability, also in terms of tissue generation or formation.

#### 3.4.4. Endodontics: Hydrogels for Pulp Regeneration

Endodontics procedure is defined as a biologically based system designed to replace defected dentin, root structure and complex pulp-dentin cells. During the development of an embryonic tooth, a cranial neural crest develops dental epithelium and ectomesenchymal cells originating from dental epithelium to give rise to dental papilla. During embryogenesis, differentiated ectomesenchymal cell aggregation or odontoblasts secrete primary dentin surrounding the dental pulp. The dental pulp is soft tissue placed in tooth core containing nerve and blood vessels subjected to chemical, thermal, mechanical and bacterial insults. Generally, infection in the pulp chamber due to bacterial infection may cause serious damage including tooth fracture and failed root canal treatment (RCT) [164].

Endodontic tissue regeneration is based on the principle of conventional regenerative medicine. The regenerative endodontics involves research concerned with the use of growth factors, stem cells, organ tissue culture as well as tissue engineered matrix. Generally, highly structured mechanically strong 3-D scaffolds provide excellent support to the cells used in any area of body including bone where a tailored scaffold is needed to provide physical support. However, in case of root canal treatment or more specifically for pulp complex regeneration, strong scaffold cannot be placed to deliver structural support to the tooth. Thus, engineered pulp tissue is administered as 3-D soft templates such as polymer hydrogel.

Injectable hydrogels are considered as outstanding biomaterials for therapeutic cell and bioactive molecules delivery for guided dental tissue engineering, especially for pulp complex regeneration applications. Hydrogels possess tuneable tissue-like characteristics, controlled degradation and release features, easy adaptability in clinical settings for minimal invasive surgery and shape conformation ability, i.e., to conform 3-D defects upon gelling. Endodontic tissue engineering is based on the principle of conventional regenerative medicine. In regenerative endodontics, injectable hydrogels have shown feasibility of delivering dental pulp stem cells, supporting template-like enamel derivatives and growth factors (FGF-2, BMP-1, SDF-α1) to support dentin pulp complex formation. Controlled release of growth factors from dentin is an important defence mechanism against dental injuries [165]. These growth factors are trapped in dentin template at the time of dentinogenesis and are retained at the site as quarantined” molecules.

Hydrogels made of natural polymers are highly biocompatible, inexpensive, readily available and easy to tailor by applying simple and cost-effective techniques. They have been investigated as injectable scaffolds worldwide for dentin-pulp complex regeneration. Some of the natural polymers include chitosan, collagen, gelatine, alginate and hyaluronic acid. The applications of these polymers in pulp regeneration are described briefly below.

Feng at al. [166] demonstrated that the granular 3-D chitosan scaffolds fabricated by freeze-drying technique support neural differentiation of dental pulp stem cells (DPSCs). Chitosan hydrogel exhibits good conductivity and forms suitable template for cell attachment, survival and differentiation. Moussa et al. [167] have also investigated the mechanical characteristics of collagen and found that its intrinsic mechanical property is sufficient for dental pulp regeneration and can further be improved by incorporation calcium phosphate ceramic. Ishimatsu et al. [168] examined the effect of controlled release of fibroblast GF-2 as different doses from gelatine hydrogel to regenerate dentin-pulp complex. The released doses of this specific growth factor influenced calcified tissue structure regenerated inside dentin defects as the gelatine hydrogel supported osteodentin. Fujiwara et al. [169] utilized alginate scaffolds to transplant subcutaneous HDPCs in nude mice back. After six weeks post implantation, subcutaneous formation of radio-opaque calcified bodies was noticed. The isolated ondoblast-like cells lead to the initiation of dentin-like hard tissue formation. On the other hand, Inuyama et al. [170] investigated the effects of hyaluronic acid matrices as endodontics from amputated dental pulps for rat molars in vivo. The dentin defects treated with hyaluronic acid hydrogels exhibited a cell-rich recognizing tissue structure denoting the possibility of further study of this polymer for dental pulp regeneration.

On the other hand, synthetic hydrogels possess finely tuned characteristics—biodegradability and high reproducibility with little batch to batch variation. The commonly used synthetic hydrogels for dental pulp regeneration: polylactic acid (PLA) and polyglycolic acid are described briefly. Scaffolds made of these polymers are conductive for seeding stem cells such as SHED and DPSCs. Stem cells were found to differentiate into odontoblast-like cells on these hydrogels, leading to endothelial cell stimulation to generate tissues resembling dental pulp and dentin. Mooney at al. [171] demonstrated a technique to fabricate new pulp-like tissue structures by utilizing cultured cells and synthetic ECM. Fibroblasts were seeded on polyglycolic acid-based materials and the cells were found to adhere, proliferate and differentiate to form neo-tissue after 60 days of culture to mimic natural pulp. Buurma et al. [172] reported that HDPs and gingival fibroblasts attached to polyglycolic acid matrix survived when implanted subcutaneously in mice. These transplanted cells secreted collagen type I and cellular fibronectin.

Cordeiro et al. [173] investigated importance of vascularised soft connective pulp-like tissue and new tubular dentin formation when SHED was firmly seeded in PLA hydrogels. The matrices were incorporated in the tooth slice and transplanted subsequently in nude mice. The resulting tissue exhibited almost similar architecture of natural dental pulp. Besides, supramolecular hydrogels are next-generation biomaterials to be used for dental pulp regeneration. These materials are 3-D entities prepared synthetically from crosslinking agents that bond via hydrogen bond or Vander Waals interaction to form desired hydrogel. The injectable supramolecular hydrogels can be potentially used for dental tissue engineering, owing to their efficiency in delivering therapeutics like bioactive molecules and cells in a sustained and controlled manner.

#### 3.4.5. Endodontics: Platelet Rich Fibrin (PRF) for Pulp Regeneration

Platelet rich fibrin (PRF) is a fibrin matrix in which the platelet cytokines and cells are wedged to serve as a resorbable matrix following their release after a stipulated time. PRF was used initially in oral implantology, but, it has also shown wide application in pulp regeneration. PRF is considered as an appropriate scaffold material and a promising tool for regenerative endodontics due to its ability to control differentiation of stem cells and deliver growth factors in sustained and controlled manner. Platelet rich plasma (PRP)/PRF combined with collagen membranes was used extensively for periodontal ligament and alveolar bone regeneration as guided tissue in periodontics and oral surgery [174].

Yang et al. [175] demonstrated the importance mixing of fibrin glue with PRF as matrix seeded with dental bud cells for dental regeneration applications. PRF is rich in tissue growth factor-β (TGF-β) and platelets derived growth factor (PDGF) and has major roles in angiogenesis and hard tissue regeneration. Moreover, dental bud cells grown on PRF/PRP templates were found to regenerate a complete tooth along with enamel, dentin, cementum, pulp, blood vessels as well as PDL. Jayalakshmi et al. [176] utilized PRF along with β-TCP to treat periapical cyst and significant and progressive radiographic bone regeneration was noticed. Keswani et al. [177] reported that PRF might serve as an ideal template for the revascularization of necrotic pulp of immature permanent teeth as PRF is rich in growth factors, thus enhancing proliferation and differentiation of cells for tissue in-growth. Bains et al. reported the application of PRF to manage iatrogenic perforation of pulpal floor in furcation area of the mandibular root (molar) due to biocompatible and autologous nature of PRF and MTA [178].

Shivashankar et al. [179] reported necrotic pulp featured tooth revitalization and open apex by utilizing PRF. Huanget et al. [180] investigated the biological effects of PRF on human dental pulp cells. PRF resulted in enhanced proliferation of dental pulp cell and osteoprotegerin (OPG) expression in a time dependant manner. PRF also resulted in upregulated alkaline phosphate (ALP) activity. The only disadvantage lying with PRF is its handling and manipulation to be placed into root canal.

#### 3.4.6. Photopolymerizable Materials

Photopolymerizable and the biodegradable materials are getting popular with extended use and applications in relation to the field of tissue engineering such as bone, teeth, liver, and cartilages. The technology of photo-initiated polymer making has allowed the creation of injectable photopolymerizable biomaterials that is for dental and other tissue engineering applications [181]. As such the process resulted in the creation of cells, and the conductive environment for growth that facilitates the fabrication of the scaffold materials hence facilitates the complex structures. The elements that result from this process range from the purely synthetic products such as the polyethylene glycol, and others made wholly of natural polymers such as hyaluronic acid [182]. These natural and synthetic polymers get mixed or reacted with specific functional groups that are targeted to cause degradation on the enzymatic or the hydrolytic bond that make up the different polymer crosslinks or the backbone [181]. Photopolymerizable biomaterials are used to develop injectable biomaterials used for the production of scaffolding with multifaceted structures and deliver growth factors and cells for dental tissue engineering. The photopolymerizable biomaterials are also used to deliver molecules and drugs. Polymeric materials offer scaffolding to control the evolution and development and of three-dimensional tissues in dental or other tissue engineering [183,184].

#### 3.4.7. Hybrid Bioceramics Materials Hydrogels

Various scaffolds such as polymeric scaffolds, natural polymers, synthetic polymer, self-assembling materials, hydrogels, extra-cellular matrix, and the bioceramics materials are used in the dental field as the materials for pulp tissue regeneration [10,185,186,187]. These materials can be used to form soft connective tissues and new dentin during dental tissue engendering. In addition, peptides materials are the most preferred materials for tissue engineering in dentistry due to their rotational design properties. The peptide sequence plays a vital role in enabling control of the stiffness of the material; it induces mineral nucleation and introduces the antibacterial activity, which promoted tissue regeneration after dental surgery [162].In addition, the modern approaches of creating or generating pulp in dental tissue engineering depends on the type of the scaffold materials such as collagen, chitosan, polyesters, and hydroxyapatite [188].

Scaffolds can be used in dental pulp regeneration. Moreover, combining stem cells with the inductive scaffold materials will optimize the regeneration of dentine pulp [151]. The use of self-assembling hydrogels of peptides is an excellent example of the smart scaffold materials, for dental tissue engineering applications because these materials can be easily customized and modified into desired shapes from matrices. Additionally, the authors proposed the use of bio injectable engineering materials in dental tissue engineering [189,190].

Nojoomi et al. [191] found that the most common biomaterials used in dental tissue engineering include the natural polymers, synthetic polymer, self-assembling materials, hydrogels, extra-cellular matrix, and the bioceramics materials. In another study, Mao et al., [44] found that the use of growth factors and the new biomaterial scaffolds, which orchestrate the homing of host endogenous cells, is the best cell transplantation approach that facilitates or accelerates dental tissue regeneration in the dental tissue engineering field. On the contrary, Orellana et al. [131] argued that complex dentin-pulp engineering should focus on considering and controlling the aspects such as the vascularization, the growth factors involvement, matrix degradation, the interactions between the cells and the matrix, the factor of mineralization, and the aspects of contamination control.

#### 3.4.8. Using Calcium Phosphate Cements (CPCs) as Scaffold Material for Dental and Bones Regeneration

Calcium phosphate cements (CPCs) are used as injectable scaffolds for dental tissue regeneration engineering [192,193]. Simon, Guthrie and Wang [194] investigated how cement of calcium phosphate (CPC) can be applied through injection to harden the in situ, and found that CPCs can be used for craniofacial and dental application, especially in dental tissue engineering. Calcium hydroxide is broadly used for direct pulp capping in the current clinical dental tissue engineering. Calcium hydroxide is used for the regeneration of hard tissues and dental pulp tissues upon dental injuries. Moreover, calcium hydroxide plays an important role in pulp capping therapy [195]. The current development of new processing technologies in the production of calcium phosphate cement scaffolds focuses on biological responses by putting more emphasis on the improvement of biological interactions of CPCs with tissues and cells and tissues, and the use of CPCs in dental as well as bone tissue engineering. Calcium phosphate cements are increasingly used in clinical uses, for example, in tissue engineering, because of their advantages, which include mouldability, injectability, osteoconductivity and bioactivity features [193,196,197,198,199,200].

In another study, Thein-Han et al. [201] examined the use of calcium phosphate cement mixed with bio functional agents or factors and seeding of stem cells focused on treating dental repair and the repair of the cranial facial bones. The study focused on incorporating biofunctional factors or agents to CPC and to study the activity of the “human umbilical cord mesenchymal stem cells (hUCMSC) seeding on biofunctionalized CPC for osteogenic differentiation. The authors developed a method that used five different types of bioactive agent. The agents used in the study were RGD (Arg-Gly-Asp) peptides, human-derived fibronectin (FN), extracellular Geltrex matrix, a concentrate of human platelet, and an engineered fibronectin-like protein polymer. Additionally, the study used five different biofunctionalized CPC scaffolds. The fabricated structures include; CPC-Fn, CPC-FEPP, CPC-RGD, CPC-platelets, and CPC-Geltrex. The study measured the activity hUCMSC proliferation, attachment, synthesis, and osteogenic differentiation. The study finding revealed that the hUCMSC on bioactivated CPCs indicated improved cell attachment, actin fibre expression, proliferation, mineral synthesis, and osteogenic differentiation. In addition, the new CPCs developed included CPC-Fn, CPC-FEPP, CPC-RGD, CPC-platelets that have improved properties compared to traditional materials and the biofunctionalized CPC material greatly facilitates bone regeneration for dental and bone applications. Based on their findings, Thein-Han, Liu and Xu, [201] recommended that calcium phosphate cement (CPCs) can be used as an injectable scaffold biomaterial for bones regeneration, and to regenerate dental pulp or tissues in dentistry applications or engineering.

## 4. Hydrogel Properties versus Cross-Linking Density

The polymer chains present in hydrogels are crosslinked with each other either physically or chemically. Minute environmental condition changes such as pH, temperature, presence of enzyme or ionic species, electric signal might elicit fast and reversible changes in the hydrogel system. These changes usually take place at microscopic level as change in conformation, precipitation and water content of hydrogel [202]. Few factors that are correlated with hydrogels versus crosslinking density are as follows:

### 4.1. Swelling

The swelling kinetics of hydrogels are classified as Fickian (diffusion controlled) and non-Fickian (relaxation controlled) swelling kinetics. The swelling kinetics is practically diffusion controlled when water diffusion into hydrogels occurs at faster rate than polymer chain relaxation. The crosslinking ratio is one of the best factors to influence swelling of hydrogels. The higher the crosslinking ratio, the more the crosslinking agent is interlinked in the hydrogel network. The highly crosslinked hydrogel system has a more organized and compact structure and tends to swell in small amounts as compared to hydrogels with low or without crosslinking ratios. Generally, crosslinking hinders the polymer chain mobility, thus, the swelling ratio is decreased. The chemical structure of hydrogel also influences its swelling ratio [203].

### 4.2. Mechanical Properties

The mechanical strength of hydrogel is increased with the increase in crosslinking density, leading to the formation of stronger and stiffer gels. The increase in crosslinking density also results in lowering of percentage (%) elongation of the hydrogel. Hence, the hydrogel turns brittle in nature. Many researchers have demonstrated co-polymerization with H-bonding increment within the hydrogel system to attain desirable mechanical properties.

### 4.3. Biocompatible Properties

Synthetic hydrogel polymerization contains toxic elements derived from organic solvents, emulsifiers, and stabilizer. Mostly, crosslinkers present immense challenges for in vivo biocompatibility. Natural polymers are considered to have better biocompatibility over synthetic polymer-based hydrogels. However, natural polymers incorporated with synthetic crosslinkers or initiators for polymerization possess similar toxicity concerns such as that of the synthetic hydrogels [204].

## 5. The Application of Electrospun Nanofiber in Dental Fields

The electrospinning technique is one of the versatile techniques, which is very popular because of its broad applications in the dental and medical areas. The electrospinning technique is focused on its applications in tooth regeneration, healing and preventing diseases such as dental caries. The materials used with the electrospun technique possess unique characteristics such as a higher surface area to volume ratios, have improved cellular interactions, and better protein absorption, and thus the enhanced absorption that leads to good binding sites and enhanced cells reception [120,205]. Others found that the use of various flexible biodegradable polymers (for example, PEG (poly (ethylene glycol), as well as polyesters including polycaprolactone (PCL), poly (D,L-lactide-co-glycolide) (PLGA), poly(glycolic acid) (PGA), poly(lactic acid) (PLA), as well their copolymers), as scaffolds (nanofibrous scaffolds) increases enhances regeneration of dental tissues [53,206,207].Furthermore, Kim et al., [208] found that nano-structured implants improved dental restoration and regeneration efficiencies as a result of the structural similarities to that of an ECM (natural extracellular matrix).

Compared to collagen nanofibrous scaffolds made through integrin-intermediated procedure, the use of polycaprolactone (PCL) and collagen-based nanofibrous scaffolds made via electrospinning integrating bioactive glass nanoparticles to regenerate dentin-pulps enhances odontogenic and growth differentiation from human dental pulp stem cells (hDPSCs) [208,209,210,211]. Electrospinning produces a complex geometry of nanofibrous scaffolds used to effectively regenerate dentin-pulp complexes. The dentin-pulp complex generation aims at restoring both physical and mechanical characteristics of the tooth structure in dental engineering. Electrospun nanofibers significantly control the discharge/release of biomolecule therapeutics (for example, growth factors), and adaptation with adhesive biomolecules (such as RGD (arginylglycylaspartic acid) sequence and fibronectin), and also enhance the regeneration of dental tissues. In addition, electrospun nanofibers offer mechanical functionalities and features biologically that play a vital role in dental uses. Electrospun nanofibers can, therefore, be used to carry dental resultant stem cells for optimal dental tissue regeneration for dental engineering applications. This is because electrospun nanofibrous scaffolds are easy to fabricate, and have features such as fibre alignment and control over scaffold size [212,213,214,215].

**Figure 6:**
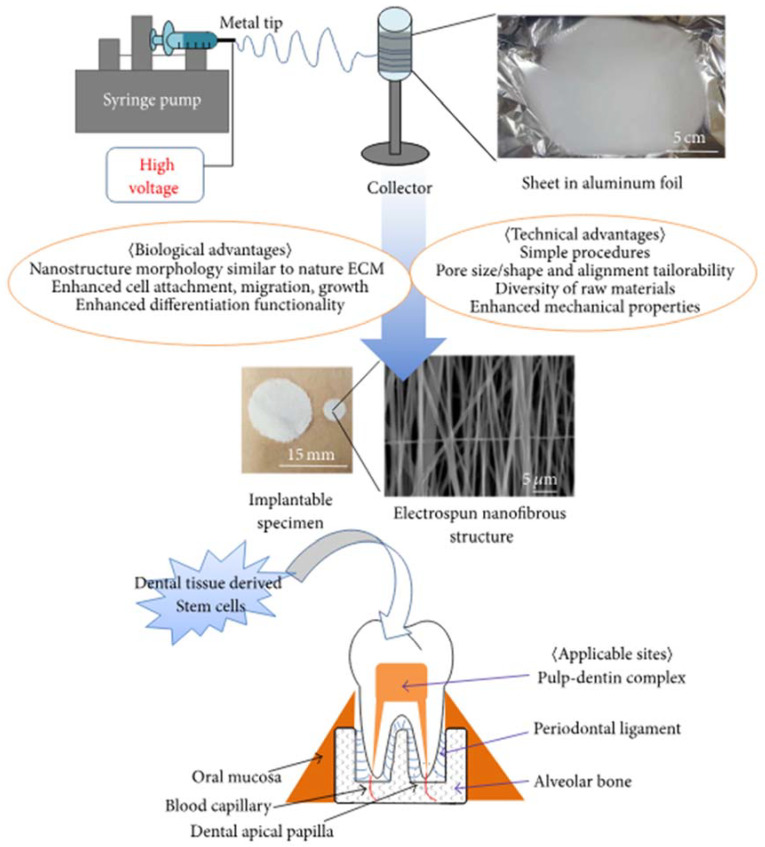
Dental tissue regeneration using polycaprolactone (PCL) electrospun nanofibrous scaffold biomaterials. Adapted from Seo et al. [216].

Electrospun nanofibers such as polymeric nanofibers or bioceramic nanoparticle-integrated nanofibers are applied in dentistry. In addition, their flexibility and nanostructure have resulted in highly promotive cell homing behaviours, leading to enhanced dental health [214]. In addition, electrospun nanofibrous scaffold biomaterials including polycaprolactone is used to regenerate dental tissues in dental tissue engineering, as shown in Figure 6 below.

Kuchler-Bopp et al. [217] examined the use of nanomaterial and the different application in tissue engineering and found that nanomaterials are successfully solving the dental problems through tissue engineering. On the other hand, nanofibers are preferred due to the ability to induce cell invasion and promoting proliferation. In this regard, nanofibers are very attractive because they closely resemble the extracellular matrix and have large pores thus suitable for bones, cartilages, and tooth regenerations in tissue engineering.

Aurrekoetxea et al. [38] found that dental pulp stem cells is an alternative choice for dental pulp regeneration and the dental pup stem cells can be used to solve dental pulp regeneration and its related problems. People suffer from various dental defects due to diseases, trauma, and tumours. As such, there has been the development of nanomaterial, which has excellent features of biomimetic characteristics and great physiological and chemical properties. The properties of the nanomaterials are very crucial in solving dental-related problems in tissue engineering because they facilitate cell growth and stimulate the regeneration of dental pulp or tissues [68,218,219]. Li et al. [220] added that the most common types of nanomaterial include the nanofibers, nanoparticles, nanosheets, and nanotubes. Furthermore, nanoparticle and nanofibers are effectively used in the delivery system to transport bioactive factors or agent in tooth and bone regeneration. In addition, nanoparticle and nanofibers have an improved control regarding the release of the agent that causes degradation and accelerate the of tissue regeneration [220]. Nanostructured scaffolds regulate cell mitigation, differentiation, as well as proliferation, and offer closer structural support approximation for the cells leading to the formation of functional tissues in tissue engineering applications. Nanomaterials are also used in repairing and regenerating tissues in clinical applications or engineering [221,222].

## 6. Summary

Biomaterials are mostly indispensable for dental application, mainly for periodontitis treatment, endodontic pulp-dentin complex reconstruction and overall tooth regeneration. Soft biomaterials such as hydrogels may have the potentialities to serve as cell encapsulating matrix. However, a tooth should assume appropriate anatomical dimension and shape to function in the dentition [223]. An appropriate biomaterial in tooth generation should possess the following characteristics:

- Non-toxic, biocompatible and can undergo biodegradation;
- Allows biofunctionality of cells such as odontoblasts, ameloblasts, fibroblasts, cementoblasts, vascular cells and neural endings;
- Fabrication of natural or synthetic polymers or combination of both as hydrogels (preferably injectable hydrogels);
- Reasonable shelf life, can be sterilized and stored in clinical settings and key approach for clinicians.

Generally, cell doctrine, scaffolds and biological signals are the principle guiding elements for tissue engineering. The bioactive scaffolds consist of embedded bioactive molecules/cells to serve the purpose. Transplantation and homing of cells are the scientific approaches of tissue regeneration. The biomolecules such as proteins, peptides and RNA/DNA can be infused in vivo to establish new environment to promote dental regeneration.

On the other hand, cell free approaches utilizing bioactive scaffolds, cells as well as growth factors from dentin behave like chemoattractants for endogenous stem cells that reside in the periapical region or in the surrounding healthy pulp tissue. These cells are expected to adhere, proliferate and differentiate to give rise to new tissue, and thus the physiological tissue condition of dental pulp is mimicked. Periodontal regeneration remains a seriously challenging task as the existing regeneration techniques have not achieved predictable periodontal tissue regeneration in humans. The synergistic combination of tissue engineering and nanomedicine has led to a remarkable progress in the periodontal tissue engineering field. Several polymers such as chitosan, collagen, alginate and hyaluronic acid as hydrogels have been investigated to promote successful guided tissue regeneration [224]. One of the most challenging aspects of establishing regenerative endodontic therapy is to determine the optimization and integration of different component procedures to generate an outcome of regenerated pulp-dentin complex. Comprehensive research programs and clinical applications are required for the possible future regenerative endodontic procedures development [75]. However, these studies need more investigation regarding in vitro and in vivo experiments to highlight their potentialities in this promising field.
